# Optimal timing and sequence of combining stereotactic radiosurgery with immune checkpoint inhibitors in treating brain metastases: clinical evidence and mechanistic basis

**DOI:** 10.1186/s12967-023-04089-4

**Published:** 2023-04-05

**Authors:** Wentao Tian, Xianjing Chu, Guilong Tanzhu, Rongrong Zhou

**Affiliations:** 1 Department of Oncology, Xiangya Hospital, Central South University, No. 87 Xiangya Road, Kaifu District, Changsha, 410008 China; 2 Xiangya Lung Cancer Center, Xiangya Hospital, Central South University, Changsha, 410008 China; 3 National Clinical Research Center for Geriatric Disorders, Xiangya Hospital, Central South University, Changsha, 410008 China

**Keywords:** Cancer combination therapy, Immune checkpoint inhibitors, Brain metastases, Stereotactic radiosurgery

## Abstract

Recent evidence has shown that immune checkpoint inhibitors (ICIs) are efficacious for treating brain metastases of various primary tumors. However, the immunosuppressive tumor microenvironment and the blood–brain barrier (BBB) or blood-tumor barrier (BTB) essentially restrict the efficacy of ICIs. Stereotactic radiosurgery (SRS) can be a powerful ally to ICIs due to its trait of disrupting the BBB/BTB and increasing the immunogenicity of brain metastases. The combination of SRS + ICI has shown synergy in brain metastases in several retrospective studies. Nevertheless, the optimal schedule for the combination of SRS and ICI in brain metastases is yet to be determined. In this review, we summarized the current clinical and preclinical evidence on the timing and sequence of SRS + ICI to provide insight into the current state of knowledge about this important area in patient care.

## Background

Metastatic brain tumors occur in approximately 20% of patients with malignant tumors [1]. The existence of brain metastases often indicates poor quality of life and survival with a 2 year survival rate of patients with brain metastases less than 10% in general [2]. The evolving anti-tumor systemic management has improved the survival of patients to a certain degree [3]. However, due to the pharmacokinetic blocking effect of therapeutic agents by the blood–brain barrier (BBB) or the blood-tumor barrier (BTB), and differentiated tumor microenvironment (TME) from the primary tumor, these treatments have rather limited efficacy in suppressing the progression of brain metastases [4]. Still, around one-third of patients with brain metastases die of intracranial progression eventually [4, 5]. Thus, treatments for brain metastasis are yet to be improved.

In the past decade, immune checkpoint inhibitors (ICIs) have primarily reshaped the landscape of anti-tumor treatments for various solid tumors [6]. The mechanism of ICIs is restoring anti-tumor immunity by blocking immune checkpoints, such as cytotoxic T-lymphocyte antigen 4 (CTLA-4), programmed cell death protein 1 (PD-1), and programmed cell death protein ligand 1 (PD-L1) [6]. Also, several meta-analyses support that ICI monotherapy is efficacious for metastatic brain tumors [7, 8]. However, in the included studies of these meta-analyses, only patients with treatment-naïve metastases were enrolled, and the vast majority of prospective studies excluded patients with symptomatic brain metastases [7, 8]. Yet, only 20% of the patients benefited from immunotherapy [7]. The efficacy of ICIs for other patients needs further investigation.

Stereotactic radiosurgery (SRS) has been broadly applied in brain metastases of various cancers [9]. Owing to its modest improvement in patients’ survival and lower chance of causing neurocognitive toxicities compared with whole-brain radiotherapy (WBRT), the American Society for Radiation Oncology recommended SRS as a preferred treatment or an alternative to WBRT for patients with newly diagnosed single or multiple brain metastases who have a good performance status [10]. And the American Society of Clinical Oncology recommended SRS alone for patients with 1 to 4 unresected brain metastases (small cell lung cancer excluded) and patients with 1 to 2 safely resected brain metastases for treating remaining intracranial disease [11]. SRS also has excellent potential to be combined with ICIs because of its trait to temporarily “open up” the BBB/BTB and cause the death of tumor cells which can induce an inflammatory microenvironment with enriched infiltration of antigen-presenting cells (APCs) and cytotoxic T lymphocytes (CTLs) [12, 13]. Although previous meta-analyses denied the clinical advantage of radiotherapy (RT) + ICI compared with ICI monotherapy [7, 14], potential bias may exist due to the retrospective nature of the included studies and baseline differences in patients’ status of the two groups, because only patients with symptomatic brain metastases receive RT + ICI in clinical practice and those who receive ICI monotherapy usually have better baseline performance statuses. As for stage III non-small cell lung cancer (NSCLC), patients who started ICI treatment < 14 days after RT gained significantly better benefits than those who received ICIs ≥ 14 days after RT in a randomized controlled trial [15]. However, the optimal SRS + ICI schedule for brain metastases, especially the sequence of and the interval between SRS and ICI, remains controversial. The understanding of the brain microenvironment after RT may help us guide the development of more effective strategies for brain metastases treatment.

In this review, we aim to summarize recent clinical and preclinical evidence concerning the combination of SRS and ICI in treating brain metastases. From that summary, we aim to provide a perspective on how to sequence and time the two therapies to improve the clinical outcomes of patients with brain metastases.

## Therapeutic mechanisms of ICIs in brain metastases

The BBB can keep out harmful macromolecular substances, playing an important role in maintaining intracranial homeostasis. It consists of tightly connected endothelial cells, mural cells, astrocytes, and basement membranes [16]. The presence of primary or metastatic brain tumors can harm the tight junctions of the BBB and relatively increase the permeability to fuel growth and invasion, and the BBB is often referred to as the BTB in the context [17]. Although the brain was initially considered to be “immune-privileged” due to the isolating capacity of BBB/BTB, newer evidence has shown that brain immunity is just limited rather than “silent” [18]. Intracranial antigens can be recognized by local APCs or translocated to cervical lymph nodes, where the APCs present these antigens to T cells and activate them [19]. Meanwhile, identical antigens released from primary tumors can be recognized and presented to T cells by APCs in adjacent lymph nodes [20]. Activated T cells in the blood, when entering the intracranial metastatic site, get involved in a series of leukocyte-endothelial cell interactions, enabling CTLs to roll and crawl along the vessels to reach a most permissible region for diapedesis [21, 22]. The extravascular CTLs then secrete matrix metalloproteases to decompose dystroglycans in the glia limitans for the final traversal through the BBB/BTB [22, 23]. However, due to chronic tumor antigen exposure, intracranial tumors can send an “off” signal to the CTLs by the binding of immune checkpoint proteins to their ligands. Blocking the interaction between the checkpoints and the ligands by an ICI (anti-PD-L1, anti-PD-1, or anti-CTLA-4) allows the CTLs to regain activity and recover their ability to kill the tumor (Fig. 3A).

The BBB/BTB has a limited permeability and only allows the penetrations of small and medium molecules [16]. Though ICIs have promising efficacy in treating brain metastases of melanoma and NSCLC, they are generally large molecules (146 kDa-149 kDa) that are not likely to cross the normal BBB and may hardly penetrate the BTB [24–27]. Even so, nivolumab, an anti-PD-1 agent, was detected in cerebrospinal fluid (CSF) of treated melanoma patients with leptomeningeal metastases. In the study, the investigators found that the CSF nivolumab concentration was 35–150 ng/mL, and the CSF/serum ratio of nivolumab concentration was 0.88–1.9% [28, 29]. Van Bussel et al. [29] proposed that the transportation was mediated by the FcRn receptors on the resided macrophages in the epithelial layer of the blood-CSF barrier, which could bind to serum nivolumab, induce endocytosis, and transport and release it to the CSF. However, the efficacy of ICIs on brain metastases is commonly believed to be based on not the direct penetration of ICIs through the BBB/BTB but the relatively autonomous trafficking of CTLs through the BBB/BTB and the moderately disrupted BTB, as mentioned above [18, 21].

Besides the immune barrier effect of the BBB/BTB, brain resident cells, such as microglia and astrocytes, also exhibit immunomodulatory effects in brain microenvironment. Microglia are resident macrophages of the central nervous system originating from the yolk sac and represent the most abundant immune cell population in brain [30]. A recent single-cell analysis has revealed considerable heterogeneity among microglia in the TME, ranging from a homeostatic to reactive phenotype continuum [31]. The homeostatic subpopulation with a ramified morphology possesses phagocytic abilities, which can enhance the therapeutic effect of ICI by presentation of tumor antigens to T cells and leading to activation of CTLs [32]. However, proliferating microglia with amoeboid morphology can inhibit the therapeutic effect of ICIs by contributing to an immunosuppressive TME [32]. Chronic IFN-γ activation in microglia has been linked to an immunosuppressive program, as demonstrated by ex vivo coculture studies. Guldner et al. [33] found that microglia in brain metastases had elevated expression of CXCL10, V-domain immunoglobulin suppressor of T cell activation, and PD-L1, which eventually result in recruitment of malfunctional T cells. Furthermore, cytotoxic T cells' production of granzyme B and IFN-γ is reduced after coculture with proliferating microglia. Proliferating microglia may also promote the depletion of CD8 + T cells. Additionally, activated microglia, along with recruited macrophages, can release a wide array of growth factors and cytokines that support tumor cell proliferation and angiogenesis [34]. Likewise, although the functions of astrocytes in brain tumors vary across subsets, some subgroups of astrocytes exhibit anti-inflammatory properties and may contribute to the immunosuppressive microenvironment [35]. Heiland et al. [36] revealed that astrocytes overexpressed interleukin-10 and transforming growth factor β (TGFβ) in company with microglia or macrophages, and the anti-inflammatory cytokines could further lead to resistance to immunotherapy and radiotherapy [37, 38]. Another subset of astrocytes characterized by high expression levels of the immune checkpoint PD-L1 and activation of the immunomodulatory factor STAT3 was identified in the peritumoral area, where they may potentially act as a barrier against anti-tumor T lymphocytes [39]. Interestingly, the presence of these phosphorylated-STAT3 immunosuppressive astrocytes is induced by tumor and microglia cells [39]. Additionally, reactive astrocytes have been shown to upregulate immunosuppressive and tumor-promoting molecules in microglia and macrophages, thus establishing a positive feedback loop between these cells and TAMs [36]. Hence, the bidirectional crosstalk between astrocytes and microglia plays a crucial role in shaping the immunosuppressive microenvironment in brain tumors.

Moreover, favorable ICI efficacy requires adequate checkpoint expressions and an immune-supportive microenvironment for both primary tumors and brain metastases. Some tumors exhibit primary resistance to ICIs or develop secondary resistance to ICIs during treatment [40]. The mechanisms of ICI resistance are rather knotty and yet to be explored, which mainly include the lack of checkpoint expression, T cell exclusion, impaired interferon signaling, antigen loss, and defective tumor antigen presentation. [41]. Previous studies have revealed a relatively equivalent level of PD-L1 expression between the primary tumor and the paired brain metastases despite noticeable temporal and spatial heterogeneities [42, 43]. This phenomenon suggests that brain metastases originating from ICI-resistant primary tumors are also unlikely to respond to ICIs. However, brain metastases are commonly characterized by much lower immune cell infiltrations and higher proportions of immunosuppressive cells compared with primary tumors, which largely limit the efficacy of ICIs in brain metastases [44]. Our research team investigated transcriptional profiles of 70 brain metastases lesions and 12 samples of paired lung adenocarcinoma and brain metastases, and we found that brain metastases presented an immunosuppressed TME compared with the primary tumor, manifested in inhibition of immune-related pathways, low expression of immune checkpoint, decreased infiltration of CD8 + T cells and cytotoxic lymphocyte, increased proportion of suppressive M2 TAMs [43]. Efforts have been made to transform this “cold tumor” phenotype into a “hot tumor”, including targeting transforming growth factor β, indolamine 2,3-dioxygenase, and tumor-associated macrophages (TAMs), etc*.* [18].

In summary, ICIs can restore the cytotoxic ability of CTL by inhibiting the interaction between the checkpoint and its ligand. Nonetheless, the therapeutic efficacy of ICIs may be hindered by several factors, including the limited permeability of the blood–brain barrier, primary resistance, as well as the immunosuppressive microenvironment within the brain. To our knowledge, SRS is one of the most promising options to fuel intracranial anti-tumor immunity, and we will discuss it below.

## Clinical efficacy of SRS + ICI in treating brain metastases

**Fig. 1:**
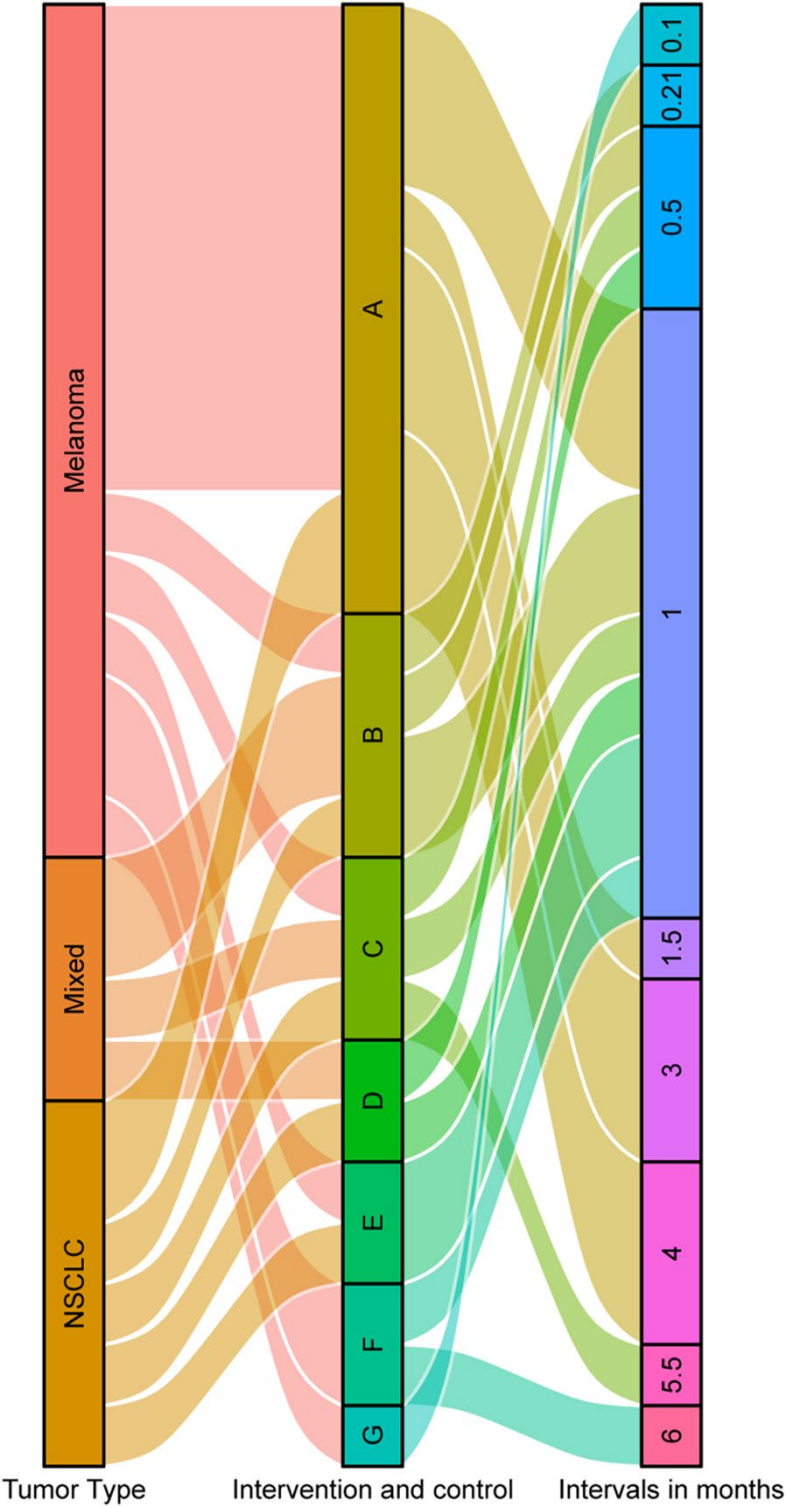
Graphic summary of studies on the combination therapy of SRS and ICI for brain metastases by tumor types, intervention/control arms, and intervals between SRS and ICI defined as “concurrent”. **A**: Concurrent SRS + ICI vs. SRS; **B**: Concurrent SRS + ICI vs. non-Concurrent SRS + ICI; **C**: Concurrent SRS + ICI vs. SRS after ICI; **D**: Concurrent SRS + ICI vs. SRS before ICI; **E**: Concurrent SRS + /before ICI vs. SRS after ICI; **F**: Non-concurrent SRS + ICI vs. SRS; **G**: Concurrent SRS + ICI vs. Concurrent WBRT + ICI

**Fig. 2:**
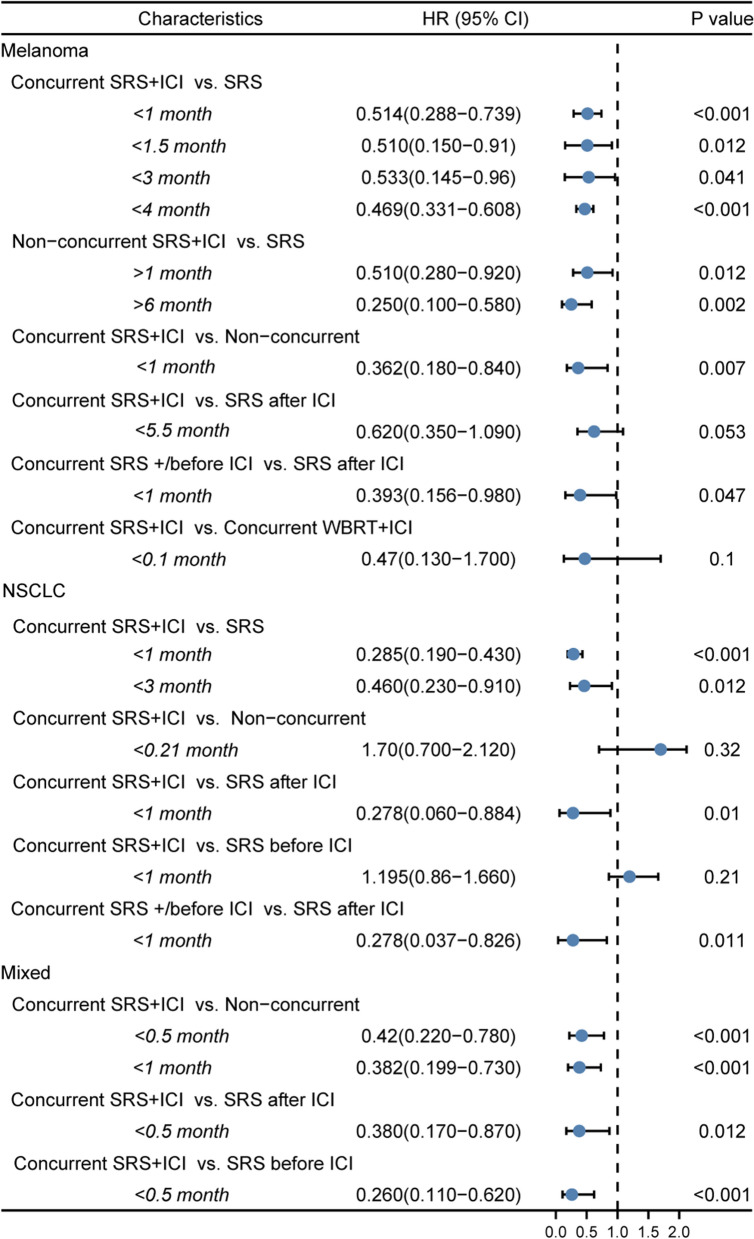
Forest plot of hazard ratios for overall survival in groups by tumor types, intervention/control arms, and intervals (months) between SRS and ICI defined as “concurrent”

In order to identify clinical studies on the combination of SRS and ICI in treating brain metastases, we searched PubMed using terms of “brain metastasis”, “stereotactic radiosurgery”, and “immunotherapy” or “immune checkpoint” and manually selected retrospective or prospective clinical studies where at least 1 arm involves SRS + ICI as a treatment for patients with brain metastases from any origins. We summarized information from all available studies on the combination of SRS and ICI in brain metastases (Table 1), including the first author, year of publication, NCT registration number (if available), type of study (retrospective/prospective), cancer type, treatments, and sample sizes (patients and lesions) of the intervention arm and the control arm, and intervals. Intervals were defined as the time interval between the first day of SRS and the most adjacent day of ICI infusion in the "concurrent" SRS + ICI treatments. The studies involved 3 classes of primary tumor types, 7 types of intervention/control combinations, and 9 definitions of intervals for “concurrent” SRS + ICI treatments (Fig. 1). In these studies, the definitions for intervals of “concurrent” SRS + ICI were quite different, ranging from 0.1 months to 6 months (Fig. 1). We demonstrated the hazard ratios for the overall survival (OS) in these studies in Fig. 2, and we also mentioned other endpoints below, such as distant brain failure, best objective response (BOR), and intracranial local control, if these data are related to the topic of this review. In short, concurrent SRS + ICI led to better outcomes compared with several controls despite varied definitions of intervals for “concurrent” SRS + ICI (Fig. 2).

**Table 1:** Characteristics of clinical studies on the combination therapy of SRS and ICI in brain metastases

Author, year	NCT number	Type of Study	Type of cancer	No. of patients (intervention)	No. of patients (control)	No. of lesions (intervention)	No. of lesions (control)	Intervention arm	Control arm	Intervals*, months	Refs
Acharya, 2017	–	Retro	Melanoma	18	38	48	121	Concurrent SRS + ICI	SRS	3	[100]
Ahmed, 2016	–	Retro	Melanoma	21	20	59	79	Concurrent SRS + ICI (anti-PD-1)	SRS	4	[101]
				25	20	73	79	Concurrent SRS + ICI (anti-CTLA-4)	SRS	4	
An, 2017	–	Retro	Melanoma	66	33	–	–	Concurrent SRS + ICI	SRS after ICI	5.5	[102]
Anderson, 2017	–	Retro	Melanoma	11	15	23	27	Concurrent SRS + ICI (anti-PD-1)	SRS	4	[103]
				20	15	31	27	Concurrent SRS + ICI (anti-CTLA-4)	SRS	4	
Choong, 2017	–	Retro	Melanoma	39	26	–	–	Concurrent SRS + ICI	SRS	1.5	[104]
Cohen-Inbar, 2017	–	Retro	Melanoma	32	14	160	72	Concurrent SRS + /before ICI	SRS after ICI	1	[105]
Diao1, 2018	–	Retro	Melanoma	18	29	59	91	Concurrent SRS + ICI	SRS	1	[106]
				25	29	160	91	Non-Concurrent SRS + ICI	SRS	1	
Diao2, 2018	–	Retro	Melanoma	23	40	–	–	Concurrent SRS + ICI	SRS	1	[107]
				28	40	–	–	Non-Concurrent SRS + ICI	SRS	1	
Kaidar-Person, 2017	–	Retro	Melanoma	29	29	–	–	Concurrent SRS + ICI	SRS	1	[108]
Kiess, 2015	–	Retro	Melanoma	15	19	–	–	Concurrent SRS + ICI	SRS before ICI	1	[58]
				12	19	–	–	SRS after ICI	SRS before ICI	1	
				15	12	–	–	Concurrent SRS + ICI	SRS after ICI	1	
				15	31	–	–	Concurrent SRS + ICI	Non-Concurrent SRS + ICI	1	
Knisely, 2012	–	Retro	Melanoma	27	50	–	–	SRS before/after/concurrent ICI	SRS	–	[109]
				16	11	–	–	SRS before ICI	SRS after ICI	–	
Lanier, 2019	–	Retro	Mixed	101	170	–	–	SRS before/after/concurrent ICI	SRS	–	[110]
Martins, 2020	–	Retro	Melanoma	28	–	–	–	Concurrent SRS + ICI	–	2.25	[111]
Mathew, 2013	–	Retro	Melanoma	25	33	99	99	SRS before/after/concurrent ICI	SRS	–	[112]
Murphy, 2019	–	Retro	Melanoma	–	–	36	54	Concurrent SRS + ICI	Non-Concurrent SRS + ICI	1	[45]
Patel, 2017	–	Retro	Melanoma	20	34	–	–	Concurrent SRS + ICI	SRS	4	[90]
Qian, 2016	–	Retro	Melanoma	33	22	–	–	Concurrent SRS + ICI	Non-concurrent SRS + ICI	1	[46]
Silk, 2013	–	Retro	Melanoma	17	16	–	–	Non-Concurrent SRS + ICI	SRS	6	[113]
Skrepnik, 2017	–	Retro	Melanoma	8	17	–	–	Concurrent SRS + ICI	Non-Concurrent SRS + ICI	1	[47]
Stokes, 2017	–	Retro	Melanoma	93	376	–	–	Concurrent SRS + ICI	SRS	4	[114]
Williams, 2017	NCT01703507	Pro	Melanoma	11	5	22	30	Concurrent SRS + ICI	Concurrent WBRT + ICI	0.1	[115]
Yusuf, 2017	–	Pro	Melanoma	18	22	59	108	Concurrent SRS + ICI	SRS	3	[116]
				7	11	21	38	SRS after ICI	SRS before ICI	3	
				12	6	41	18	Concurrent SRS + ICI	Non-Concurrent SRS + ICI	1	
Le,2022	–	Retro	Mixed	–	–	53	424	Concurrent SRS + ICI	Non-Concurrent SRS + ICI	1	[48]
				–	–	79	398	Concurrent SRS + ICI	Non-Concurrent SRS + ICI	2	
				–	–	92	385	Concurrent SRS + ICI	Non-Concurrent SRS + ICI	3	
Scoccianti,2021	–	Retro	NSCLC	100	50	–	–	Concurrent SRS + ICI	SRS	1	[54]
				90	10	–	–	Concurrent SRS + ICI	Non-Concurrent SRS + ICI	0.21	
Cabanie,2021	–	Retro	Mixed	13	46	14	79	Concurrent SRS + ICI	Non-Concurrent SRS + ICI	0.21	[51]
				35	24	40	53	Concurrent SRS + ICI	Non-Concurrent SRS + ICI	0.5	
				59	–	103	–	Concurrent SRS + ICI	–	1	
Enright,2020	–	Retro	NSCLC	33	44	64	68	Concurrent SRS + ICI	SRS	3	[117]
Weingarten,2019	–	Retro	Mixed	7	50	–	–	Concurrent SRS + ICI	Non-Concurrent SRS + ICI	1	[118]
				45	5	–	–	SRS before ICI	SRS after ICI	3	
Chen,2018	–	Retro	Mixed	28	51	–	–	Concurrent SRS + ICI	Non-Concurrent SRS + ICI	0.5	[53]
				28	22	–	–	Concurrent SRS + ICI	SRS after ICI	0.5	
				28	29	–	–	Concurrent SRS + ICI	SRS before ICI	0.5	
Ahmed,2017	–	Retro	NSCLC	13	4	35	14	Concurrent SRS + /before ICI	SRS after ICI	1	[119]
Hubbeling,2018	–	Retro	NSCLC	14	5	–	–	Concurrent SRS + ICI	SRS after ICI	1	[55]
				12	23	–	–	Concurrent SRS + ICI	SRS before ICI	1	
Schapira,2018	–	Retro	NSCLC	8	5	21	13	Concurrent SRS + ICI	SRS after ICI	1	[120]
				8	24	21	51	Concurrent SRS + ICI	SRS before ICI	1	
Kotecha,2019	–	Retro	Mixed	–	–	564	439	Concurrent SRS + ICI	Non-Concurrent SRS + ICI	5	[49]
				–	–	367	636	Concurrent SRS + ICI	Non-Concurrent SRS + ICI	1	
Khan,2021	NCT02858869	Pro	Mixed	25	–	68	–	Concurrent SRS + ICI	–	0.1	[121]
Li,2021	NCT02696993	Pro	NSCLC	13	–	–	–	Concurrent SRS + ICI	–	0.25	[122]
Wong,2021	NCT02978404	Pro	NSCLC	22	–	–	–	Concurrent SRS + ICI	–	0.5	[123]
Abdulhaleem,2022	–	Retro	NSCLC	80	235	369	522	Concurrent SRS + ICI	SRS	1	[124]
Koenig,2019	–	Retro	Mixed	27	70	359	221	Concurrent SRS + ICI	Non-Concurrent SRS + ICI	1	[52]

^*^ Intervals were defined as the time interval between the first day of SRS and the most adjacent day of ICI in the “concurrent” SRS + ICI treatments

*Retro*  retrospective, *pro* prospective, *NSCLC* non-small cell lung cancer, *SRS * stereotactic Radiosurgery, *ICI *immune checkpoint inhibitor

Studies aiming to compare concurrent SRS + ICI with non-concurrent SRS + ICI generally showed that concurrent SRS + ICI significantly improved local control of patients with brain metastases compared with non-concurrent administrations. Murphy et al. [45] identified patients with metastatic melanoma who received SRS within 30 days of receiving an ICI infusion (pembrolizumab, nivolumab, and/or ipilimumab) as those who received “concurrent” SRS + ICI. The multivariate analysis showed that concurrent timing of SRS + ICI was an independent predictor of patients’ regional progression-free survival [hazard ratio (HR) = 0.17, P < 0.0001] compared with the non-concurrent schedule [45]. Likewise, in another study by Qian et al. [46], the SRS + ICI combination was identified as “concurrent” if SRS was given within 4 weeks away from the beginning or end of ICIs, and the results demonstrated a sharper trend of reduction in metastatic melanoma lesion volume of the concurrent group compared with that of the non-concurrent group at 1.5 months, 3 months, and 6 months (P < 0.0001). Also, in the study by Skrepnik et al. [47], concurrent SRS + ipilimumab (≤ 30 days) significantly improved the regional brain control (75% vs. 23.5, P = 0.03) and prolonged median CNS progression time (not reached vs. 5.7 months; P = 0.02) for patients with intracranial melanoma metastases compared with the non-concurrent group (> 30 days), though this advantage failed to be embodied in OS. Additionally, Le et al. [48] supported that concurrent SRS + ICI (≤ 30 days) significantly decreased distant brain failure of patients with melanoma and NSCLC brain metastases compared with non-concurrent SRS + ICI or no ICI (HR = 0.15; 95% CI 0.05–0.47, P = 0.0011). Kotecha et al. [49] more specifically defined “concurrent” SRS + ICI as those with an interval ≤ 5 half-life of the ICI and “immediate” SRS + ICI as those with an interval ≤ 1 half-life, and the results revealed that the “immediate” schedule met a superior BOR of − 100% while the “concurrent” group met a BOR of − 67%. Moreover, Yang et al. [50] conducted a meta-analysis including 9 studies to compare concurrent SRS/WBRT + ICI (interval ≤ 1 month) with sequential SRS/WBRT + ICI in NSCLC patients with brain metastases, and the results showed that the concurrent schedules significantly improved intracranial local control (HR = 0.19; 95% CI 0.09–0.42; P < 0.001). However, Cabanie et al. [51] added that the time-lapse between immunotherapy and SRS was not a significant predictor of local control. They reported a 76%, 76%, and 83% 1-year local control rate for patients with an interval of less than 7 days, an interval between 1 and 2 weeks, and an interval of more than 2 weeks, respectively.

In contrast, the extracranial and survival benefits patients with brain metastases receive from “concurrent” SRS + ICI seemed to correlate with the definitions on time intervals. The study by Qian et al. [46] showed that the difference in OS between the concurrent (interval ≤ 4 weeks) and non-concurrent groups was not significant (concurrent vs. non-concurrent, median 19.1 months vs. 9.0 months, P = 0.0691). Nevertheless, Koenig et al. [52] also defined “concurrent” SRS + ICI as those with the interval within 4 weeks in patients with brain metastases of various cancers, but the results showed significantly better OS (multivariable HR = 0.57; 95% CI 0.33–0.99; P = 0.044) and lower extracranial failure rate (multivariable HR = 0.60; 95% CI 0.42–0.87; P = 0.007) compared with the non-concurrent therapy. Likewise, the aforementioned meta-analysis by Yang et al. [50] showed that concurrent SRS/WBRT + ICI (within 4 weeks to 1 month) significantly prolonged OS compared with the sequential administrations of SRS/WBRT and ICIs (HR = 0.39; 95% CI 0.16–0.97; P = 0.043). Narrowing the intervals between SRS + ICI can lead to consistent conclusions. When narrowing the defined interval of “concurrent” SRS + ICI to less than 2 weeks in patients with brain metastases in NSCLC, melanoma, and renal cell cancer, Chen et al. [53] showed an OS benefit of concurrent SRS + ICI compared with non-concurrent SRS + ICI (non-concurrent vs concurrent HR = 2.40, P = 0.006) on multivariate analysis. Moreover, when the definition of “concurrent” SRS + ICI was narrowed to less than 1 week, the research of Scoccianti et al. [54] upheld that the concurrent group had a longer OS, and the time interval between SRS and ICIs had no impact on the toxicity.

Taken together, concurrent SRS + ICI typically leads to better local control of patients with brain metastases than that of nonconcurrent schedules. Whereas it is noteworthy that although the intervention arms of these studies are all defined as “concurrent”, different intervals between SRS and ICI are likely to affect the outcomes of patients with brain metastases. There is a trend that more narrowed definitions of the time intervals result in more favorable survival benefits, suggesting that shorter intervals between SRS and ICIs lead to better clinical outcomes.

## Impact of the timing on the safety of SRS + ICI

The timing and sequence can be important factors in influencing the safety of SRS + ICI. However, contradictory results have been observed in the impact of the interval of SRS + ICI. Retrospective studies with relatively large samples have shown that SRS + ICI does not increase the rates of adverse events in patients with brain metastases compared with SRS alone, and the chance of radiation necrosis is rather low [49, 53, 55, 56]. Chen et al. [53] reported no increase in CNS toxicity or immune-related adverse events in the concurrent SRS + ICI (interval < 2 weeks) group compared with the noncurrent group (30% vs. 32%) in patients with brain metastases from various primary tumors based on a median follow-up of 9.2 months. Kotecha et al. [49] reported similar 12-month cumulative chances of radiation necrosis (3.2% vs. 3.5%) in the patients treated with immediate ICI (interval ± 1 half-life of the ICI) and all patients treated with concurrent or non-concurrent SRS + ICI. Nevertheless, some contradictory results suggested that, compared with SRS alone, SRS + ICI increased the likelihood of symptomatic necrosis within 4 years after SRS [57]. Koenig et al. [52] revealed that concurrent SRS + ICI (interval < 4 weeks) led to a higher risk of adverse radiation events (HR = 4.47, 95% CI 1.57–12.73, P = 0.005) compared with non-concurrent SRS + ICI based on a maximum follow-up of 36 months. Kiess et al. [58] also reported an increased chance of grade 3–4 adverse events in the concurrent group compared with the non-concurrent group based on a maximum follow-up of 50 months. The contradictions can be caused by distinct durations of follow-up of these studies because radiation-related or immune-related adverse events can occur even after 1 year from radiation [57]. For example, delayed radiation-induced vasculitis leukoencephalopathy related to SRS of brain metastases could be observed in 9 to 18 months after treatment [59].

In short, concurrent SRS + ICI may increase the incidences of adverse events compared with nonconcurrent administrations from the perspective of long-term follow-up, while RCTs with a large sample size and long follow-up are needed to draw a final conclusion.

## Mechanisms of synergy in concurrent SRS + ICI in treating brain metastases

### The immunostimulatory effect of SRS on brain metastases

**Fig. 3:**
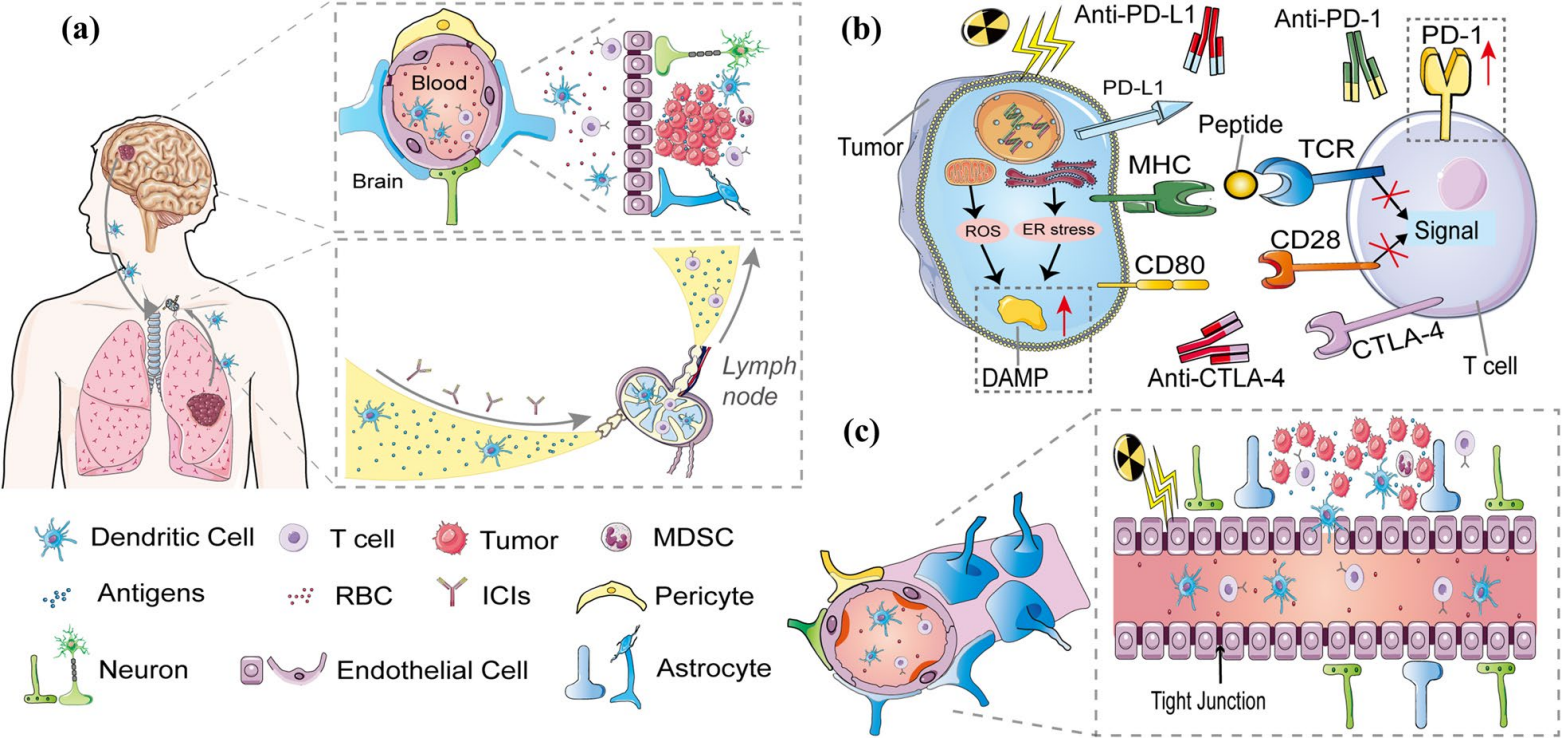
**A** APCs first identify antigens from intracranial and primary lesions, then translocate to cervical or adjacent lymph nodes, where the APCs activate T cells by presenting these antigens. Meanwhile, ICIs activate exhausted T cells by blocking the interaction between the checkpoints and the ligands. The BBB is a physical barrier made of endothelial cells with tight cell-to-cell junctions. The extravascular CTLs finally traverse through the BBB through a series of leukocyte-endothelial cell contacts and matrix metalloprotease secretion and enter the brain metastatic location. **B** Reactive oxygen species (ROS) and endoplasmic reticulum (ER) stress produced by irradiated tumor cells promote the exposure or release of damage-associated molecular patterns. On irradiation of tumor cells, PD-L1 expression is seen to have significantly increased. **C** Due to the radio-responsiveness of both the surrounding oligodendrocytes and endothelial cells, irradiation can mildly but effectively disrupt the BBB/BTB and enhance permeability. Additionally, radiation enhanced the proportion of dendritic cells and activated T cells in brain metastases

Radiation-induced immunogenic cell death (ICD) and subsequently enhanced anti-tumor immunity have received massive attention in the era of immunotherapy (Fig. 3B) [60]. Besides directly killing tumor cells by breaking double-strand DNA, radiation leads to the formation of reactive oxygen species and endoplasmic reticulum stress in tumor cells, which causes exposition or secretion of damage-associated molecular patterns (DAMPs), mainly calreticulin (CRT), heat-shock proteins (HSPs), high mobility group box 1 (HMGB1), and adenosine triphosphate, and the release of tumor-associated antigens and tumor-specific antigens [61]. The interactions between the DAMPs and their receptors initiate the recruitment and activation of APCs, especially dendritic cells (DCs), which is a prerequisite for the cross-presentation of tumor-associated antigens and tumor-specific antigens to CTLs [61–63]. Additionally, cytosolic damaged DNAs activate the DNA-sensing cGAS-STING pathway, which consequently leads to the secretion of type I interferon (T1IFN) [64]. And T1IFN correlates with enhanced crossing-priming capacity of DCs to CTLs, increased intratumor infiltration of CTLs, and cell-killing functions of the CTLs [65]. Moreover, ionizing radiation increases the expressions of major histocompatibility complex class I, Fas death receptor, and checkpoints expression on tumor cells, improving the anti-tumor immunity and sensitizing tumor cells to ICI treatment [66].

The duration of this effect is a critical concern in RT combined with ICI. Kim et al. demonstrated that the radiation-induced trafficking of the mannose-6-phosphate receptor to the cell surface enhanced the efficacy of ipilimumab, and this effect peaked within 3 days after irradiation and normalized over 7–14 days [67]. Dovedi et al. [68] demonstrated a significant decrease in PD-1 expression on both CD4^+^ and CD8^+^ T cells at day 7 after the last dose of low-dose fractioned radiation in mouse models. Gameiro et al. [69] exposed MDA-MB-231 cells to 10 Gy ^137^Cs radiation, and the results showed that the membrane CRT increased within 24 hours, but the peak of CRT was not reached due to the short follow-up of the study. And Huang et al. [70] showed that CRT exposition was time-dependent, and the level increased within 48 h in several tumor cell lines. Gorin et al. [71] established that increased Hsp70 and HMGB1 release started 24–48 hours after irradiation and lasted over 72 hours in murine colon carcinoma and murine melanoma cell lines. In contrast, Apetoh et al. [72] observed that HMGB1 was released 18 hours after X-ray irradiation in mice implanted with EG7 thymoma cells but did not detect a conspicuous exposition of HSPs. Notably, HMGB1 serves as a double-edged sword in anti-tumor immunity, as an acute increased level of HMGB1 results in the enhancement of ICD. In contrast, a lasting elevation of HMGB1 leads to immunosuppression and facilitates malignant development [73]. The interaction between HMGB1 and toll-like receptor 4 induced the expression of a non-classical type I human leukocyte antigen (HLA) molecule, HLA-G, in glioma, which assists in the immune escape of the tumor [74]. Also, the co-culture of esophageal squamous cell cancer-derived HMGB1-containing exosomes and mononuclear cells for 3 days resulted in the expansion of immunosuppressive PD1^+^ M2 TAMs [75]. Additionally, HMGB1 can enhance the immunosuppressive effect both by inducing the differentiation and activation of regulatory T cells (Tregs) [76–78] and by promoting the proliferation and survival of myeloid-derived suppressor cells [79, 80]. These results stress the importance of applying timely ICI around SRS to avoid the immunosuppressive phase.

It is also notable that radiation can lead to immunosuppressive effects that hamper the efficacy of ICIs. First, radiation can kill immune cells per se and change the composition of immune cells in the TME [81]. To be specific, Kachikwu et al. [82] showed that the proportion of immunosuppressive Tregs increased in response to radiation due to stronger resistance to radiation of Tregs than that of other lymphocytes. Second, radiation can reconstruct the immune microenvironment via various chemokines and cytokines [83]. After radiation, tumors have elevated expression of hypoxia-inducible factor 1 (HIF1), which consequently induces secretions of VEGF-A, TGFβ, and monocyte colony-stimulating factor (M-CSF) [84]. Radiation-induced release of CCL2 recruits monocytes into the TME, and M-CSF and TGFβ polarize these cells to the immunosuppressive M2 phenotype [84]. Also, overexpressed VEGF-A recruits MDSCs and facilitates Treg proliferation [84]. In summary, radiation has both immunostimulatory and immunosuppressive impacts on TME, which emphasizes the significance of counteracting the immunosuppression by immunotherapy to amplify the efficacy of treatment.

### The disrupting impacts of SRS on the BBB/BTB

The BBB/BTB raises one of the critical concerns in administrating systemic therapeutic agents to treat brain tumors, as intracranial drug delivery is commonly limited by the tight junctions in the BBB/BTB, leading to insufficient permeations of several systemic anti-tumor drugs [17]. Irradiation, especially high-dose irradiation, can temporarily but effectively disrupt the BBB/BTB, which increases its permeability due to the radio-responsiveness of both endothelial cells and surrounding oligodendrocytes [17, 85]. The disruption is characterized by both elevated paracellular and transcellular transport [17, 85]. The increased permeability partially explains radiation-induced central nervous system (CNS) toxicity, such as edema, but may also creates conditions for improved drug delivery and immune cell infiltration (Fig. 3C).

Here arises one of the crucial questions about SRS-ICI combination therapy—how long does this hyperpermeability last, and when does it reach its peak? Teng et al. used dynamic contrast-enhanced MRI images of 30 patients with brain metastases to investigate BBB opening patterns from pre-RT to one-month post-RT, and the lesions were classified as low- or high-permeability based on pre-RT transfer constant (K^trans^) [86]. The results showed that the permeability of baseline-low-permeability tumors increased over time while that of baseline-high-permeability tumors decreased over time, leading to the conclusion that systemic therapy should be conducted within 2–4 weeks after RT [86]. However, the follow-up of this study is rather short. As an indirect phenomenon, the volume of brain edema develops within 6 months and lasts for months or even years after SRS or WBRT [87]. A recently published meta-analysis summarizing the clinical and preclinical impact of conventional photon RT on BBB permeability revealed that there were increased permeabilities in all acute (< 1 month), early delayed (1–6 months), and late-delayed (> 6 months) follow-up categories [88]. Furthermore, there was no significant difference in permeability improvement among these three groups (p = 0.46) [88]. Still, significant heterogeneity (I^2^ = 99%, 96%, and 94%, respectively) existed in all of the included studies, which affected the reliability of the results to a certain degree [88].

### The half-life of ICIs and the timing of SRS + ICI

The half-life of ICIs is one of the essential factors when determining the optimal timing of combining ICIs with SRS. The half-life of ipilimumab (anti-CTLA-4), nivolumab (anti-PD-1), and pembrolizumab (anti-PD-1) are 15 days, 25–26.7 days, and 27 days, respectively [89]. This could partially explain why SRS + ICI failed to bring benefits to patients whose interval between SRS and the contiguous dose of ICI is comparatively long in some clinical studies [90].

Taken together, these preclinical results present us with a trend to apply ICIs as soon as possible but after 24–72 hours post-radiation to meet a high permeability of BBB/BTB, the most substantial immunostimulatory effect of SRS, and a higher plasma concentration of ICIs. However, most of these studies were conducted extracranially, and the dose and type of radiation varied, which might not represent the actual and accurate situation of the combination of SRS and ICI in brain metastases.

### The optimal treatment design of SRS + ICI in treating brain metastases

Treatment design plays a crucial role in the success of SRS + ICIs in the clinic. It involves careful planning, and execution of the SRS and ICIs to maximize its therapeutic effects while minimizing adverse effects. The radiation dose and fractionation and the sequence of SRS and ICIs are critical factors in the design of this combined treatment.

The radiation dose is an essential parameter that affects both the local tumor control and the systemic immune response. An ideal radiation dose will provoke inflammatory tumor cell death and activate the anti-tumor T-cell responses via APC maturation [91]. Moreover, the translocation or secretion level of DAMPs seemed to correlate with the radiation dose positively [92]. However, excessively high doses can cause damage to surrounding healthy tissues and organs, for example, brain edema develops within 6 months and lasts for months or even years after SRS or WBRT [87]. Vanpouille ‘s study showed that Trex1 could be induced by radiation doses above 12–18 Gy, regardless of cancer type. While Trex1 could turn off RT-driven immune responses by degrading dsDNA and the subsequent cGAS/STING activation [93, 94]. Therefore, it is crucial to balance the dose to maximize the immune response while minimizing the risk of adverse effects.

The fractionation refers to the number of radiation fractions delivered. By Irradiating the mice engrafted with the B16 melanoma cells for 15 Gy × 1 fraction or 5 Gy × 3 fractions, Lugade et al. [95] proved that the single-fractioned radiation increased the antigen availability and the number of tumor-specific T-cells secreting IFN-γ in the tumor-draining lymph node to a more considerable extent than the multi-fractionated RT did. The Fluctuations of permeability of BBB also correlate with the fractionation. Single high-dose irradiation leads to rapid changes while multi-fractionated RT leads to slow ones [13, 96]. However, the optimal fractionation may vary depending on tumor types and locations and should be individualized based on each patient's specific condition.

The sequence of SRS + ICI for brain metastases has been a topic of significant interest in recent years and, however, remains a topic of debate. Our meta-analysis reported that the odds ratio of distant brain failure rate of SRS-before-ICI and SRS-after-ICI was 0.67. Though there was no statistical difference, it shows the potential benefit of SRS-before-ICI strategy. Moreover, Krummel et al. [97] retrospectively identified that the SRS-before-ICI group had superior survival compared with the SRS-after-ICI group. They demonstrated that most of the deregulated genes raised in the RT-before-ICI group were involved in apoptotic signaling and were crucial modulators of activated T-cell signaling. Buchwald et al. [91] recommend RT-before-ICI therapy as well. He reckoned that SRS may obliterate the freshly infiltrated and reinvigorated T-cell reaction in the RT-after-ICI group. In contrast, RT will stimulate naïve T-cell differentiation, and T-cells apoptosis may be avoided in the RT-before-ICI group [91]. However, the sequence of treatment may be influenced by the location and size of the brain metastases. Whether the brain metastases are symptomatic is another concern in the sequence of treatment. Timely surgical resection or RT (SRS or WBRT) is recommended for patients with symptomatic lesions to manage the symptoms, while patients with asymptomatic metastases usually undergo systemic therapies and observation. Further research is needed to determine the optimal sequence of treatment for different patient populations and tumor types.

In summary, treatment design plays a critical role in optimizing the therapeutic efficacy and safety of RT combined with ICIs and should be individualized based on each patient's specific condition.

## Conclusion

The synergy between SRS and ICIs has been one of the hottest topics in treating brain metastases over years. All of the preclinical and clinical results above showed us a trend that, after 24–72 hours post-SRS, shorter intervals between SRS and ICI indicate more favorable clinical benefits for patients with brain metastases. And single high-dose irradiations appear to cause more potent immunostimulatory effects and more rapid BBB/BTB opening than fractioned low-dose ones. However, metastases from different primary tumors have varied radio-sensitivities and neo-antigen load. Additionally, the optimal sequence and interval may vary with the specific ICI administered, as CTLA-4 and PD-1/PD-L1 antagonists have different mechanisms. Hence, the optimal timing of SRS + ICI addressed in this review remains an active inquiry, which calls for well-designed prospective studies for a reliable answer.

Besides SRS, emerging therapeutic techniques are being developed to overcome the obstacles in the ICI treatment of brain metastases. Nanomedicine can target the BBB/BTB, tumor cells, immunosuppressive cells, APCs, or T cells to boost ICD and the intracranial efficacy of ICIs [98, 99]. A series of local treatments, such as focused ultrasound, tumor-treating fields, and laser therapy, can also modulate the permeability of the BBB/BTB and may have synergy with ICIs [13]. These advances may provide a solid backstop for ICI therapy for brain metastases.

## Data Availability

Data sharing is not applicable to this article as no datasets were generated or analyzed during the current study.

## Abbreviations

APCs: Antigen-presenting cells
BBB: Blood–brain barrier
BOR: Best objective response
BTB: Blood-tumor barrier
CI: Confidence interval
CRT: Calreticulin
CNS: Central nervous system
CSF: Cerebrospinal fluid
CTLs: Cytotoxic T lymphocytes
CTLA-4: Cytotoxic T-lymphocyte antigen 4
DAMPs: Damage-associated molecular patterns
DCs: Dendritic cells
HLA: Human leukocyte antigen
HMGB1: High mobility group box 1
HR: Hazard ratio
HSPs: Heat-shock proteins
ICD: Immunogenic cell death
ICI: Immune checkpoint inhibitor
IFN: Interferon
NSCLC: Non-small cell lung cancer
OS: Overall survival
PD-1: Programmed cell death protein 1
PD-L1: Programmed cell death protein ligand 1
RT: Radiotherapy
SRS: Stereotactic radiosurgery
TAMs: Tumor-associated macrophages
TME: Tumor microenvironment
TGFβ: Transforming growth factor β
Tregs: Regulatory T cells
WBRT: Whole-brain radiotherapy
